# Biosensor‐Guided Engineering of a Baeyer‐Villiger Monooxygenase for Aliphatic Ester Production

**DOI:** 10.1002/cbic.202400712

**Published:** 2024-11-06

**Authors:** Thaleia Sakoleva, Florian Vesenmaier, Lena Koch, Jarne E. Schunke, Kay D. Novak, Sascha Grobe, Mark Dörr, Uwe T. Bornscheuer, Thomas Bayer

**Affiliations:** ^1^ Department of Biotechnology & Enzyme Catalysis Institute of Biochemistry University of Greifswald Felix-Hausdorff-Str. 4 17487 Greifswald Germany; ^2^ acib GmbH Krenngasse 37/2 8010 Graz Austria

**Keywords:** Aliphatic ketones/esters, Baeyer-Villiger monooxygenase, Biosensor, Enzyme engineering, Luciferase

## Abstract

Esters are valuable aroma compounds and can be produced enzymatically by Baeyer‐Villiger monooxygenases (BVMOs) from (aliphatic) ketone precursors. However, a genetically encoded biosensor system for the assessment of BVMO activity and the detection of reaction products is missing. In this work, we assembled a synthetic enzyme cascade – featuring an esterase, an alcohol dehydrogenase, and LuxAB – in the heterologous host *Escherichia coli*. Target esters are produced by a BVMO, subsequently cleaved, and the corresponding alcohol oxidized through the artificial pathway. Ultimately, aldehyde products are detected *in vivo* by LuxAB, a luciferase from *Photorhabdus luminescens* that emits bioluminescence upon the oxidation of aldehydes to the corresponding carboxylates. This biosensor system greatly accelerated the screening and selection of active BVMO variants from a focused library, omitting commonly used low‐throughput chromatographic analysis. Engineered enzymes accepted linear aliphatic ketones such as 2‐undecanone and 2‐dodecanone and exhibited improved ester formation.

## Graphical abstract

In this work, aliphatic esters are produced by Baeyer‐Villiger monooxygenases (BVMOs) from ketone substrates. Through an artificial enzyme cascade, esters are converted into aldehydes, which are detected by a bacterial luciferase. This set‐up presents the first genetically encoded biosensor system monitoring BVMO activity and greatly accelerated the bioluminescence‐based selection of active enzyme variants, omitting initial low‐throughput chromatographic analysis.

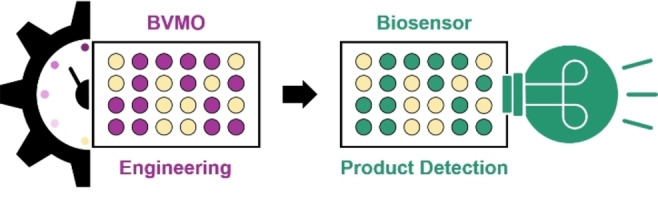

## Introduction

Over the last decades, the utilization of enzymes for applications in synthetic chemistry and biotechnology has tremendously increased.[^1^, ^2^, ^3^, ^4^] Enzymes assembled in cascade‐type reactions are of special interest. Not only do they combine the inherently high chemo‐, regio‐, and stereoselectivity of biocatalysts; reaction schemes usually do not involve hazardous chemicals, catalysts, and solvents. Furthermore, the isolation of intermediates is obsolete, which reduces experimental steps, the production of (harmful) waste, and costs.[^4^, ^5^, ^6^, ^7^, ^8^] Among industrially interesting biocatalysts are different oxidoreductases including Baeyer‐Villiger monooxygenases (BVMOs).^[4]^ These flavin‐dependent enzymes can incorporate molecular oxygen next to carbonyl groups at the expense of the hydride donor NAD(P)H, forming the corresponding esters or lactones.[^9^, ^10^, ^11^, ^12^, ^13^] BVMOs exhibit a wide substrate scope and catalyze the oxidation of aliphatic^[9]^ and α,β‐unsaturated ketones,^[13]^ (bi)cyclic ketones,[^13^, ^14^] steroids,^[10]^ as well as epoxidation reactions^[15]^ or the oxidation of heteroatoms.[^14^, ^16^] One of the main limitations of BVMOs is the so‐called *’uncoupling‘* reaction, which occurs in all monooxygenases and produces hydrogen peroxide (H_2_O_2_) at the expense of NAD(P)H, without inserting an oxygen atom into the substrate.^[5]^ While established protein engineering techniques have been employed to both expand the substrate scope of BVMOs[^5^, ^17^] and reduce uncoupling,^[18]^ the screening of variants usually relies on chromatographic methods for the (direct) detection and quantification of target products or the (indirect) photometric monitoring of NAD(P)H oxidation at 340 nm.[^18^, ^19^, ^20^, ^21^] The generation of H_2_O_2_ from the uncoupling reaction can interfere with absorbance assays for NAD(P)H, thereby, contributing to an overestimation of product formation.[^22^, ^23^] While chromatography‐based methods are low‐throughput and regularly involve time‐consuming sample preparation,[^3^, ^24^, ^25^] these bottlenecks have been partially addressed by the use of colorimetric or fluorescence‐based assays.[^7^, ^26^, ^27^] However, these strictly depend on chromo‐ and fluorogenic surrogate substrates, respectively; the conversion of linear aliphatic ketones by BVMOs, thus, cannot be followed in real‐time. In this regard, genetically encoded biosensor systems have been utilized for the detection of a variety of small molecules.[^3^, ^28^, ^29^] Biosensor systems can feature transcription factors, riboswitches, or two‐component systems as sensory parts that are coupled to the activity of reporter genes. The latter generate a measurable read‐out in the presence of the target analyte.[^3^, ^28^, ^29^, ^30^] Complementary, enzyme‐coupled devices yield read‐outs such as bioluminescence by the conversion of substrate compounds.[^24^, ^31^, ^32^] Today, biosensors are employed to facilitate the directed evolution of enzymes[^33^, ^34^, ^35^, ^36^] and the optimization of metabolic pathways through the high‐throughput (HT) detection of metabolites,[^37^, ^38^, ^39^, ^40^] amongst other biotechnological applications.[^28^, ^29^, ^41^, ^42^] While an amperometric biosensor, consisting of *Escherichia coli* (*E. coli*) whole‐cells immobilized in a polyelectrolyte membrane onto an oxygen electrode, was used to monitor BVMO activity,^[43]^ a genetically encoded biosensor system for the detection of aliphatic esters is missing.

**Figure 1:**
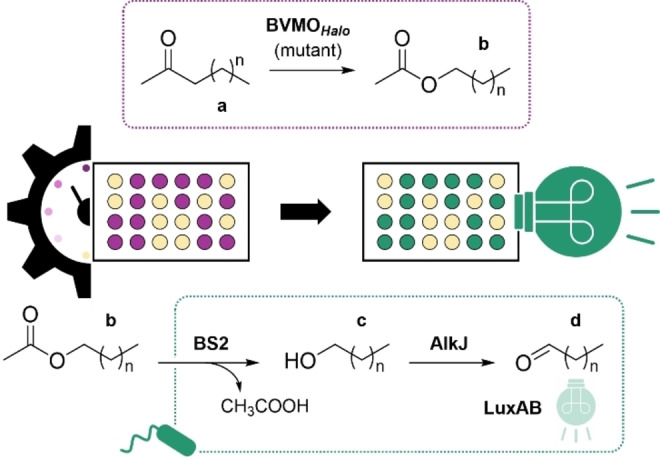
BVMO engineering and LuxAB‐based biosensor design. Aliphatic ketones (**a**) are oxidized by BVMOs. The produced esters (**b**) are hydrolyzed *in vivo* by the esterase BS2. The corresponding alcohols (**c**) are oxidized by the ADH AlkJ to aldehydes (**d**), which are converted by LuxAB, yielding the carboxylate (not shown) and bioluminescence as the read‐out. The HT detection of aldehydes enables the reliable assessment of the activity of BVMO variants in a 96‐well plate format; n=3–14. Co‐factors are omitted for clarity.

In this work, we present a biosensor‐based screening assay to determine the activity of BVMOs. Firstly, linear aliphatic ketones are converted into the corresponding esters by BVMOs. In a cascade‐type reaction, these esters are hydrolyzed enzymatically by the *Bacillus subtilis* esterase 2 (BS2), yielding a carboxylic acid and a primary alcohol. The latter is oxidized by an alcohol dehydrogenase (ADH). Ultimately, aldehyde products are detected by LuxAB, a flavine mononucleotide (FMN)‐dependent monooxygenase from *Photorhabdus luminescens* (*P. luminescens*), which emits bioluminescence upon the oxidation of aldehydes to the corresponding carboxylates (Figure 1).[^24^, ^44^] This LuxAB biosensor system has been implemented in an engineered *E. coli* K‐12 MG1655 strain with reduced aromatic aldehyde reduction activity, in the following referred to as *E. coli* RARE, for the robust and efficient detection of aldehydes produced enzymatically.[^24^, ^44^, ^45^]

Herein, the Baeyer‐Villiger reaction is catalyzed by the recently characterized BVMO_*Halo*_ from *Halopolyspora algeriensis*.^[9]^ Considering the industrial relevance of oxidative enzymes such as BVMOs, their tailoring by protein engineering has been of particular interest. The presented engineering campaign targeted the active site of BVMO_*Halo*_ and utilized the biosensor‐based HT detection of reaction products to select promising enzyme variants. Furthermore, the applied enzymatic cascade yields aliphatic medium‐ and long‐chain esters and aldehydes (Figure 1), which have applications as flavorants and food additives.[^24^, ^46^, ^47^]

## Results and Discussion

### LuxAB‐Based Biosensor Assembly and Characterization

**Figure 2:**
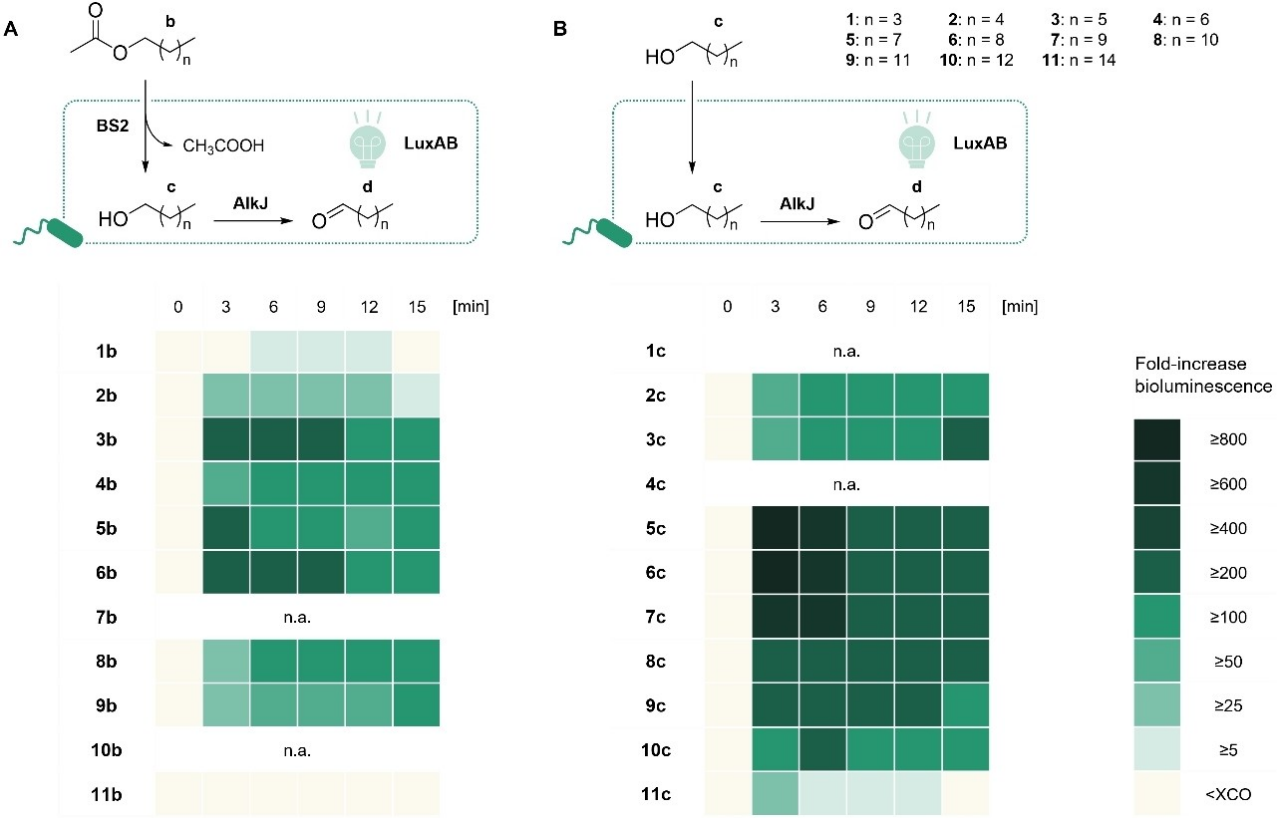
LuxAB‐based HT assays. (**A**) Aliphatic esters (**1–11 b**) are cleaved by BS2 and the corresponding alcohols are oxidized by AlkJ to aldehydes. The latter are sensed by LuxAB expressed in the same cell. (**B**) Primary alcohols (**1–11 c**) are added directly, oxidized, and the corresponding aldehydes detected as before; co‐factors are omitted for clarity. Bioluminescence signals monitored over time before (0 min) and after the addition (3–15 min) of 1 mM substrate to RCs of *E. coli* RARE (OD_600_≈10.0), co‐expressing BS2 from pET28a, AlkJ and LuxAB from pLA1. The XCO value was determined in the presence of 1 % (*ν/ν*) dimethylformamide (DMF) as described below.^[24]^ Heat‐maps show the mean fold‐increase in bioluminescence above background of biological replicates (n=3); n.a.=not available (**7 b**, **10 b**, **1 c**, and **4 c**).

In previous studies, the heterodimeric luciferase LuxAB was expressed from a streptomycin‐selectable pCDF backbone under the control of a constitutive T5 promoter.^[24]^ Detectable aldehydes were produced *in situ* by different oxidoreductases, expressed in the same cell from compatible and inducible T7 promoter‐based vectors.[^8^, ^24^, ^32^] Together, aromatic, terpenoid, and aliphatic aldehydes could be synthesized and detected in *E. coli* RARE in a 96‐well plate format.[^24^, ^29^, ^32^] In this work, the bioluminescence assay is coupled to the two‐step transformation of aliphatic ketones into primary alcohols, catalyzed by BVMO_*Halo*_ or corresponding variants and the esterase BS2 (Figure 1).^[48]^ Since the system under investigation requires the heterologous expression of multiple biosynthetically unrelated enzymes, we aimed at reducing the metabolic burden by combining the open reading frames (ORFs) encoding the *luxA* and *luxB* subunits from *P. luminescens* and *alkJ* from *Pseudomonas putida* on a single plasmid (pCDF_*luxAB::alkJ*), in the following referred to as pLA1. Therefore, we employed the sequence‐ and ligation‐independent cloning (SLIC) techniques described previously.[^49^, ^50^] Functionality of the pLA1‐based system was confirmed by bioluminescence assays using 1‐decanol (**6 c**) as the luciferin precursor (Figure S2). While the two‐plasmid system (pACYQ_*alkJ* + pCDFduo_*luxAB*)^[24]^ yielded a >1,500‐fold increase in bioluminescence after 15 min reaction time, the single plasmid system (pLA1) produced bioluminescence ∼1,000‐fold above background. The bioluminescence output was more consistent with pLA1 between independently carried out assays as suggested by a smaller standard deviation (SD) and described in the Supporting Information (SI; Figure S2). Subsequently, we introduced the esterase BS2, encoded on a pET28a vector. The bioluminescence output was recorded after adding either esters (**1**–**11 b**) or primary alcohols (**1**–**11 c**) to *E. coli* RARE resting cells (RCs), co‐expressing BS2, AlkJ, and LuxAB (Figure 2).

Comparable to previous findings, bioluminescence was emitted in response to the *in situ* production of different aldehydes.^[24]^ In this work, we could expand the detection scope to long‐chain aldehydes like hexadecanal, produced from the precursors **11 b** or **11 c** (Figure 2). Within the 15 min monitoring time, the strongest bioluminescence signals were detected for the esters heptyl acetate (**3 b**), nonyl acetate (**5 b**), and decyl acetate (**6 b**; Figure 2A), as well as the alcohols 1‐nonanol (**5 c**), 1‐decanol (**6 c**), and 1‐dodecanol (**7 c**; Figure 2B). The related ketones, which are substrates for BVMO_*Halo*_, did not increase the bioluminescence above the experimental cut‐off (XCO) values as described below. This proves that ketones do not interfere with the biosensor system (Table S3).

Consequently, the LuxAB‐based biosensor was applied to detect linear aliphatic esters, produced in whole‐cells containing BVMO_*Halo*_ wild‐type or enzyme variants.

### Engineering of BVMO_*Halo*_ and LuxAB‐Guided Screening and Selection of Variants

**Figure 3:**
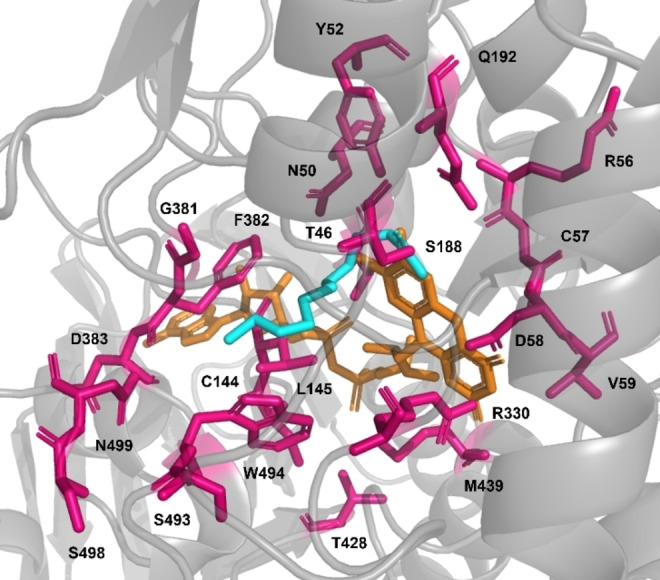
Homology model of the active site of BVMO_*Halo*_. The FAD co‐factor (orange) was placed based on the crystal structure of the *Thermobifida fusca* phenylacetone monooxygenase [PDB: 1W4X]^[56]^ due to the high sequence identity (56.7 %) and structure alignment (Root Mean Square Deviation [RMSD]=1.037 Å as calculated by PyMOL v3.0); **6 a** (cyan) was docked as described below. Amino acid residues selected for alanine scanning are shown in magenta, the backbone of BVMO_*Halo*_ in grey.

Earlier, BVMO_*Halo*_ was shown to be a linear aliphatic ketone monooxygenase.^[9]^ To determine crucial amino acid residues in the active site, contributing to the overall activity of BVMO_*Halo*_, a homology model was created by AlphaFold and used to dock 2‐dodecanone (**6 a**) as a reference substrate into the active site as described below (Figure 3).[^51^, ^52^, ^53^] Structural analysis yielded 21 active site residues, either interacting with flavin adenine dinucleotide (FAD) or the docked **6 a**. These 21 amino acids were targeted by alanine scanning, substituting wild‐type amino acids with the small amino acid alanine. Mutants were created by established site‐directed mutagenesis protocols (i. e., QuikChange^®^).[^54^, ^55^]

**Figure 4:**
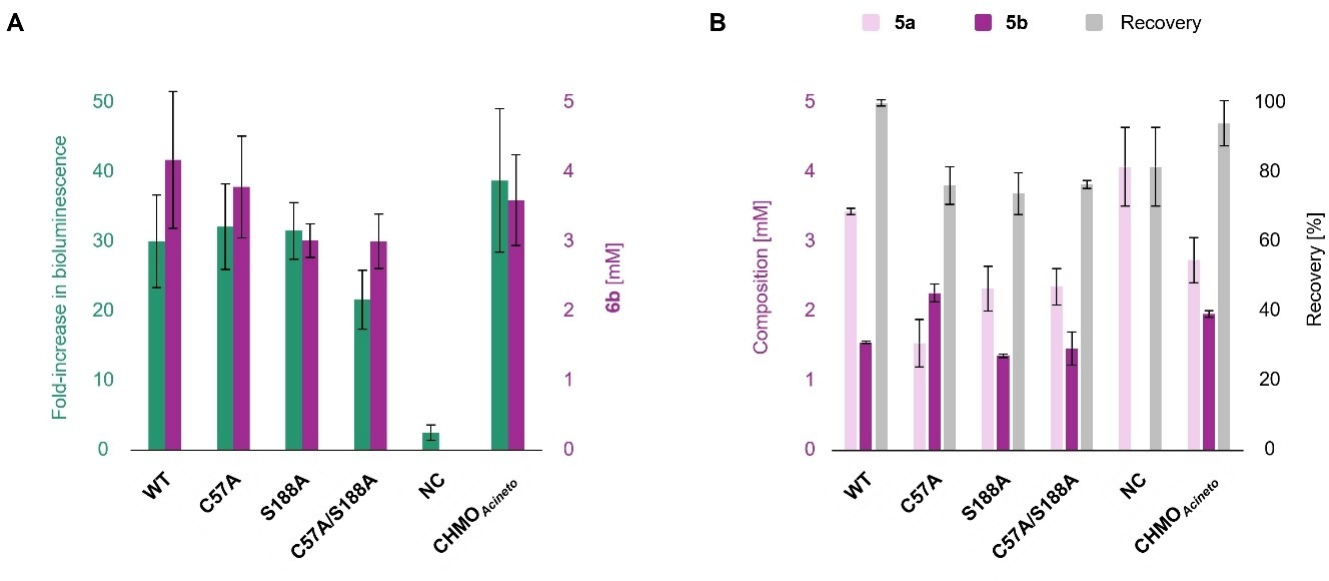
Activity of BVMOs and variants *in vivo*. (**A**) Formation of ester **6 b** from the benchmark ketone **6 a** monitored by LuxAB‐based assays (green) and confirmed by GC/FID analysis (magenta). The variants C57A, S188A, and the corresponding double‐mutant exhibited similar activities than BVMO_*Halo*_ wild‐type (WT). *E. coli* TOP10 transformed with the empty pBAD vector was used as negative control (NC) and did not convert **6 a**; the cyclohexanone monooxygenase from *Acinetobacter* sp. (CHMO_*Acineto*_) was used as additional control for the oxidation of aliphatic ketones.^[59]^ The fold‐increase in bioluminescence and the concentration of **6 b** are given as mean values ± SD of biological replicates (n≥2). Biosensing conditions are described below. Biotransformations were performed at 25 °C in *E. coli* RCs as indicated (OD_600_≈10.0); 5 mM substrate load, 5 % (*ν/ν*) DMF as co‐solvent. For time‐resolved production of **6 b** and recovery of material, see Figure S4. (**B**) Conversion of ketone **5 a** (light pink) into the desired ester **5 b** (magenta). Biotransformation conditions as in (**A**); overall conversion of **5 a** was lower than for **6 a**. The composition of reaction mixtures [mM] and recovery of material [%] are represented as mean values ± SD of biological replicates (n≥2). Reduced recoveries attributed to low solubility and/or volatility of compounds.^[24]^

Expression of individual clones of this focused library was performed from pBAD vectors in *E. coli* TOP10, applying a modified small‐scale protein production protocol in 96‐deep well plates.^[57]^ Noteworthy, BVMO_*Halo*_ production was not successful in *E. coli* RARE, probably due to the metabolization of the inducer arabinose (data not shown).[^45^, ^58^] Subsequently, cell‐free extracts (CFEs) or *E. coli* TOP10 whole‐cells containing BVMO_*Halo*_ variants were used to convert the reference ketone **6 a** into the corresponding ester **6 b**. After 24 h reaction time, biotransformation mixtures were transferred into 96‐well microtiter plates containing RCs of *E. coli* RARE, co‐expressing BS2, AlkJ, and LuxAB as before. Analysis of reaction mixtures yielded two promising BVMO_*Halo*_ mutants – C57A and S188A – that showed fold‐increases in bioluminescence comparable or even higher to the wild‐type enzyme (see Figure S3 for *in vitro* and Figure 4A for *in vivo* reactions). All other variants were in the range of the background signal. To assess the background, CFEs or RCs were prepared from *E. coli* TOP10, harboring the empty pBAD vector instead of BVMO_*Halo*_ variants.

Next, we quantified the production of **6 b** from **6 a** by calibrated GC/FID analysis. As anticipated, the variants with bioluminescence read‐outs comparable to the empty vector control had not converted the ketone **6 a** (data not shown). The variants C57A and S188A yielded 3.78±0.73 mM and 3.01±0.24 mM **6 b**, respectively, after 24 h. The BVMO_*Halo*_ WT produced 4.17±0.98 mM **6 b**. We then combined single‐point mutations, yielding the BVMO_*Halo*_ C57A/S188A double‐mutant. Again, increased bioluminescence signals correlated with BVMO activity *in vivo* (Figure 4A). To demonstrate the applicability of the biosensor system, we also investigated CHMO_*Acineto*_, which – besides activity on cylohexanone (Figure S5) – had been reported to exhibit promiscuous activity towards aliphatic compounds.[^59^, ^60^] The C57A/S188A variant and CHMO_*Acineto*_ produced 3.00±0.39 mM and 3.59±0.65 mM **6 b**, respectively, after 24 h.

Hence, these screening applications validated the functionality of the biosensor set‐up by the identification of BVMOs and engineered variants with ester‐forming activity (Figure 4).

Since the benchmark ketone **6 a** was equally well converted by BVMO_*Halo*_ wild‐type (WT) and active variants, we exemplarily investigated the acceptance of the shorter 2‐undecanone (**5 a**) and the longer 2‐tridecanone (**7 a**) over time; the corresponding aldehyde products are luciferins for LuxAB as determined in this work (Figure 2) and reported previously.^[24]^ While **7 a** was only poorly converted by the employed biocatalysts (data not shown), *n*‐nonyl acetate (**5 b**) was readily produced (Figure 4B). Although the overall conversion of **5 a** was lower than for **6 a** (Figure 4), C57A performed best and yielded 2.25±0.34 mM **5 b** – compared to 1.55±0.05 mM produced by the WT – in 24 h (1.5‐fold improvement). The S188A and C57A/S188A variants showed slightly reduced activity, CHMO_*Acineto*_ yielded 1.95±0.32 mM **5 b** according to calibrated GC/FID analysis. As before, no conversion of the ketone substrate was detected in *E. coli* TOP10 RCs harboring the empty pBAD vector (Figure 4B).

## Conclusions

In this work, we assessed the activity of two BVMOs accepting linear aliphatic ketones by a genetically encoded biosensor system. The produced esters were converted into the corresponding aldehydes through an artificial enzyme cascade (BS2, AlkJ) in *E. coli* RARE. Ultimately, aldehydes were detected by the luciferase LuxAB from *P. luminescens* (Figure 1). Straightforward implementation of the newly assembled sensor plasmid pLA1, encoding the ORFs of *alkJ* and *luxAB* on a single vector, facilitated the bioluminescence‐based identification of ester‐yielding BVMO_*Halo*_ variants under different HT screening conditions (Figure 2, Figure 4A, and Figure S3). In combination with the robust expression of enzyme variants in a 96‐well plate format^[57]^ and the initial omission of low‐throughput sample preparation and chromatography‐based analysis of reaction products, the investigated biosensor system offers great potential to accelerate enzyme discovery, engineering, and metabolic pathway optimization – beyond the tailoring of BVMOs for aliphatic ester production.[^4^, ^24^]

## Experimental Section

### General Materials, Strains and Plasmids

Unless stated otherwise, all chemicals, reagents, and solvents were purchased from Sigma‐Aldrich (Steinheim, Germany), Fluka (Buchs, Switzerland), New England Biolabs (NEB; Ipswich, MA, USA), or Merck/Millipore (Darmstadt, Germany) and used without further purification. Primers were synthesized and desalted by the Eurofins Genomics GmbH (Ebersberg, Germany). Sanger sequencing was performed by the Microsynth AG with the company's standard primers (Balgach, Switzerland). *E. coli* DH5α and TOP10 were purchased from Invitrogen. *E. coli* RARE was a gift by the Prather group but is also available from Addgene (#61440).^[45]^ Expression of BVMO_*Halo*_ (or variants) from pBAD was performed in *E. coli* TOP10 upon addition of arabinose.^[9]^ CHMO_*Acineto*_ ^[59]^ and the esterase BS2^[48]^ were produced from pET28a vectors in *E. coli* RARE. AlkJ and LuxAB were either co‐expressed from a two‐plasmid system^[24]^ or from pLA1 as described below.

### Homology Model Creation and Molecular Docking

A homology model of BVMO_*Halo*_ was generated using AlphaFold.^[52]^ Docking experiments were performed in YASARA as described above and in the SI to map the active site of BVMO_*Halo*_ and to select amino acid residues for site‐directed mutagenesis.^[53]^

### Site‐Directed Mutagenesis

Site‐directed mutagenesis was performed by using the Q5^®^ Mutagenesis Kit (#E0554S, NEB) and following the instructions by the supplier. Primers were designed with the NEBaseChanger^®^ v2.4.2 webtool (https://nebasechanger.neb.com/). PCR mixtures with a total volume of 25 μL contained 12.5 μL 2X high‐fidelity Q5 master mix, forward and reverse primer (1.25 μL each of a primer solution with a concentration of 0.5 μM; Table S1), 1 μL of plasmid template (final amount=2 ng μL^−1^), and 4 μL nuclease‐free water. Thermal cycle conditions are given in Table S1; annealing temperatures were taken from the automated primer design tool. PCR mixtures were incubated at 37 °C for 1 h after the addition of 1 μL DpnI (#10196884, NEB). Subsequently, 1 μL of the DpnI‐digested PCR mixture was diluted with 3 μL nuclease‐free water, to which 5 μL 2X KLD reaction buffer and 1 μL 10X KLD enzyme mix were added. It was gently mixed, spun down, and the resulting solution incubated at 37 °C for 30 min. The resulting solution (5 μL) was used to transform chemically competent *E. coli* TOP10 cells as described below. After growth overnight on lysogeny broth (LB) agar plates supplemented with 100 μg mL^−1^ ampicillin, plasmid DNA was isolated from a single colony, using the innuPREP Plasmid Mini Kit 2.0 (iST Innuscreen, Berlin, Germany). The desired mutations were confirmed by Sanger sequencing.

### Construction of pLA1

The ORF encoding *alkJ* together with its T7‐*lac* promoter and the ribosome binding site was amplified from pACYQ_*alkJ* and subcloned into pCDFduo_*luxAB* – downstream of the LuxAB‐coding region – through SLIC as reported previously.^[24]^ The DNA fragment containing *alkJ* was amplified with Pfu^+^ and the primer pair AlkJ‐CDF_F/R (T_a_=48.6 °C), introducing 20 bp‐overhangs complementary to the target pCDF vector. The pCDF backbone was amplified with the primers pCDF_F/R and *Opti*Taq (T_a_=48.6 °C). The preparation of PCR mixtures and optimized thermal cycle conditions are given in the SI. Purified DNA fragments (pCDF_*luxAB* ≈ 5.7 kb and *alkJ* ≈ 1.8 kb) were processed and incubated with a SLIC extract as reported before.[^24^, ^49^, ^50^] Subsequently, chemically competent *E. coli* DH5α cells were transformed with the assembly mixtures. Single colonies were selected on LB agar plates containing 25 μg mL^−1^ streptomycin. Sanger sequencing, using the primers SEQ‐AlkJ_F/R and SEQ‐mAlkJ_F/R, confirmed the correct assembly of pCDF_*luxAB::alkJ*, herein referred to as pLA1. Primer sequences are given in Table S2.

### Preparation and Transformation of Chemo‐Competent *E. coli* Cells

Prior to transformation of *E. coli* RARE, plasmids were isolated from *E. coli* DH5α or TOP10.^[24]^ Preparation of chemically competent cells and transformation by heat‐shock was followed by previously published protocols with minor modifications. Briefly, a single colony of the desired *E. coli* strain was cultivated in LB medium containing the appropriate antibiotic(s) at 37 °C with shaking (180 rpm; INFORS HT Multitron) for 12–16 h. For the selection of transformants, 100 μg mL^−1^ ampicillin (pBAD), 34 μg mL^−1^ chloramphenicol (pACYC‐derived vectors), 50 μg mL^−1^ kanamycin (pET28a), and 25 μg mL^−1^ streptomycin (pCDF‐derived plasmids) were used. For the selection of cells harboring multiple plasmids, the concentration of antibiotics was reduced by half. In baffled flasks, LB medium – supplemented with antibiotic(s) if applicable – was inoculated with 1 % (*ν/ν*) of a pre‐culture grown overnight. Main cultures were incubated at 37 °C with shaking (180 rpm) until an optical density measured at 600 nm (OD_600_) of 0.2–0.4 was reached. An aliquot (750 μL) of the resulting culture was transferred into a sterile 1.5 mL tube and centrifuged at 4,000 x g, 4 °C for 10 min. All following steps were carried out on ice under sterile conditions. The supernatant was removed and the cell pellet was resuspended in 350 μL ice‐cold 0.1 M CaCl_2_ and incubated for 15 min. After centrifugation (2,500 x g, 4 °C for 10 min), the supernatant was discarded. The cell pellet was resuspended in 50 μL ice‐cold 0.1 mM CaCl_2_. Plasmid DNA (2 μL with a concentration of 25–100 ng μL^−1^) was added. To introduce BVMO_*Halo*_ mutants generated by site‐directed mutagenesis, 5 μL of the KLD‐treated solution were used as described above. The resulting mixture was incubated for 1 h on ice. Heat‐shock was performed at 42 °C for 45 s before putting the transformation mixture back on ice for 2 min. Recovery was performed in 500 μL super optimal broth with catabolite repression (SOC) medium (2 % (*ω/ν*) tryptone, 0.5 % (*ω/ν*) yeast extract, 10 mM NaCl, 2.5 mM KCl, 10 mM MgCl_2_, 10 mM MgSO_4_, and 20 mM glucose) at 37 °C with vigorous shaking for at least 1 h. Transformants were selected on LB agar plates containing 1.5 % (*ω/ν*) agar‐agar in the presence of the appropriate antibiotic(s). As mentioned above, only half the concentration was used for the selection and subsequent cultivation of strains harboring multiple plasmids.^[24]^

### Protein Expression in *E. coli* RARE and Preparation of RCs

Protein production (CHMO_*Acineto*_, BS2, AlkJ, and LuxAB) was performed in *E. coli* RARE using LB‐based auto‐induction medium (2.5 % (*ω/ν*) LB medium, 1 mM MgSO_4_, 25 mM (NH_4_)_2_SO_4_, 50 mM KH_2_PO_4_, 50 mM Na_2_HPO_4_, 0.5 % (*ω/ν*) glycerol, 0.05 % (*ω/ν*) glucose, and 0.2 % (*ω/ν*) α‐lactose) as described previously.^[24]^ Antibiotic(s) were added as before. For the preparation of RCs, cultures were harvested by centrifugation (6,000 x g, 4 °C) for 20 min. The cell pellet was resuspended in resting cell medium (RCM; 22 mM KH_2_PO_4_, 42 mM Na_2_HPO_4_, 8.56 mM NaCl, 1 mM MgSO_4_, 0.1 mM CaCl_2_, and 1 % (*ω/ν*) glucose) until an OD_600_≈20.0 was reached. To confirm protein production, whole‐cell samples were denatured as described in the SI and analyzed by sodium dodecyl sulfate‐polyacrylamide gel electrophoresis (SDS‐PAGE; Figure S1).

### Small‐Scale Expression of BVMO_*Halo*_ Variants

Small‐scale cultivations were carried out in 96‐deep well plates (2 mL Masterblock^®^ with V‐shaped bottom; #780271, Greiner Bio‐One, Frickenhausen, Germany) as described previously with minor modifications.^[57]^ Briefly, a single colony of the desired *E. coli* TOP10 transformant was used to inoculate 400 μL LB medium containing 100 μg mL^−1^ ampicillin. It was incubated at 37 °C with shaking (>700 rpm in an orbital plate shaker; TiMix TH30, Edmund Bühler GmbH, Bodelshausen, Germany) for 16 h. For enzyme production, 800 μL of modified PSAM‐5052 medium (0.2 g L^−1^ of each amino acid as indicated below, 0.16 g L^−1^ methionine, 25 mM (NH_4_)_2_SO_4_, 50 mM KH_2_PO_4_, 50 mM Na_2_HPO_4_, 0.5 % (*ω/ν*) glycerol, 0.05 % (*ω/ν*) glucose, 0.2 % (*ω/ν*) α‐lactose, 1X trace elements, 1 mM MgSO_4_, 0.01 mM FeCl_3_, and 0.1 μM vitamin B12) supplemented with ampicillin was inoculated with 2 % (*ν/ν*) of the preculture. The 50X amino acid mixture contained (10 g L^−1^ each): sodium glutamate, aspartate, lysine‐HCl, arginine‐HCl, histidine‐HCl, alanine, proline, glycine, threonine, serine, glutamine, asparagine, valine, leucine, isoleucine, phenylalanine, and tryptophane. The 1,000X trace element solution was prepared according to Studier.^[61]^ After 5 h of incubation (37 °C, >700 rpm), 0.2 % (*ω/ν*) arabinose was added from a 20 % (*ω/ν*) stock solution. The plate was further incubated at 20 °C with shaking overnight. Cell pellets were harvested by centrifugation (3,000 x g, 4 °C for 30 min). The supernatant was discarded and the pellet re‐suspended either in RCM until an OD_600_≈20.0 was reached or in 300 μL lysis buffer (50 mM KH_2_PO_4_/K_2_HPO_4_, pH 6.5) containing 1X BugBuster^TM^ (#70921, Novagen). Lysis was performed at 20 °C for 1 h. Cell‐free extracts (CFEs) were obtained by centrifugation (3,000 x g, 4 °C for 30–40 min).

### Standard Bioluminescence Assay

The LuxAB‐based detection of aldehydes was adapted from established protocols.[^24^, ^32^] Briefly, RCs of *E. coli* RARE co‐expressing B2S (pET26b), AlkJ and LuxAB (pLA1) were prepared as before. In 96‐well plates (white, flat bottom; #655074, Greiner Bio‐One), 198 μL RCs (OD_600_≈10.0) were pipetted and the background bioluminescence measured before the addition of any analyte‐containing solution (t_0_) on a Varioskan^TM^ LUX multimode plate reader (Thermo Fisher Scientific). Afterwards, 2 μL of different organic compounds – ketones (**1**–**11 a**), esters (**1**–**11 b**), and primary alcohols (**1**–**11 c**) – were added from 100 mM stocks (prepared in DMF; 1 mM final concentration). The bioluminescence was recorded for 15 min (Figure 2; see also Figure S2). The XCO value was determined in the presence of 1 % (*ν/ν*) DMF as described previously.^[24]^ To calculate the fold‐increase in bioluminescence in response to aldehyde formation, the signal at each time point (t_x_) was divided by the background signal at t_0_.

### Biotransformation Reactions and LuxAB‐Based Evaluation

For *in vitro* reactions, 60 % (*ν/ν*) CFE, 1.25 mg mL^−1^ rhamnolipids (#R90, Sigma‐Aldrich), 1 mM **6 a** (2‐dodecanone), and 1 mM NADPH were combined in 50 mM KH_2_PO_4_/K_2_HPO_4_ (pH 6.5). Reactions were incubated at 20 °C with shaking (200 rpm) for 24 h.^[9]^ Subsequently, 52 μL of each reaction mixture were mixed with 148 μL *E. coli* RARE RCs (OD_600_≈15.0) co‐expressing BS2, AlkJ, and LuxAB. The bioluminescence was measured before (t_0_) and after the addition of reaction mixtures (up to 15 min) and fold‐increases calculated (Figure S3).

For whole‐cell biotransformations, RCs of the desired *E. coli* transformant were prepared as before and adjusted to an OD_600_≈10.0 with RCM. Ketone substrates (5 mM final concentrations) were converted at 25 °C with shaking (220 rpm; INFORS HT Multitron) for 24 h. For GC/FID analysis, samples (100 μL) were extracted as described below. Prior to bioluminescence assays, RC suspensions were snap‐frozen in liquid nitrogen and thawed to permeabilize cell membranes. It was centrifuged (8,000 x g, 15 min) and the clarified supernatant (20 μL) was transferred into 96‐well microtiter plates containing 180 μL *E. coli* RARE RCs (OD_600_≈10.0) co‐expressing BS2, AlkJ, and LuxAB. The bioluminescence was measured and fold‐increases calculated as before (Figure 4A).

### Quantification by GC/FID

Biotransformation samples (100 μL) were extracted two times with 200 μL ethyl acetate containing 1 mM methyl benzoate as internal standard (IS). After vortexing for 1 min and centrifugation (12,000 x g, 1 min), the upper organic phase was transferred into a fresh 1.5 mL tube. The extraction was repeated and the combined organic phases were dried over anhydrous MgSO_4_ or Na_2_SO_4_. Samples were analyzed on a GC‐2010 Plus (Shimadzu) equipped with a flame ionization detector (FID) and an auto‐injector (Shimadzu). Separation was achieved on a ZB5MSi column (length: 30 m; inner diameter: 0.25 mm; film thickness: 0.25 μm) from Phenomenex (Torrance, USA), using methods as reported previously.^[24]^ For quantification, the relative response factors (RFFs) of investigated compounds were determined as before.[^24^, ^62^] RFFs were used as mean values of independently prepared compound dilutions (n≥4) in ethyl acetate containing 1 mM IS (Table S7).

## Conflict of Interests

The authors declare no conflict of interest.

1

## Supporting information

As a service to our authors and readers, this journal provides supporting information supplied by the authors. Such materials are peer reviewed and may be re‐organized for online delivery, but are not copy‐edited or typeset. Technical support issues arising from supporting information (other than missing files) should be addressed to the authors.

Supporting Information

## Data Availability

The data that support the findings of this study are available from the corresponding author upon reasonable request.
