# Supplementary material for: Biosensor‐Guided Engineering of a Baeyer‐Villiger Monooxygenase for Aliphatic Ester Production
Source: Chembiochem. 2024 Nov 6;26(1):e202400712. doi: 10.1002/cbic.202400712 (PMC11727011; doi:10.1002/cbic.202400712)
Supplement: Supplementary file 1 — Supporting Information [file CBIC-26-e202400712-s001.pdf]

ChemBioChem

Supporting Information

Biosensor-Guided Engineering of a Baeyer-Villiger Monooxygenase for Aliphatic Ester Production

Thaleia Sakoleva, Florian Vesenmaier, Lena Koch, Jarne E. Schunke, Kay D. Novak, Sascha Grobe, Mark Dörr, Uwe T. Bornscheuer, and Thomas Bayer*

Table of Contents

Experimental Procedures and Additional Results **2**

Site-directed mutagenesis of BVMO$_{Halo}$ and assembly of the biosensor plasmid pLA1 **2**
Table S1 2
Table S2 3
Table S3 3

Protein expression and SDS-PAGE analysis **3**
Figure S2 3

LuxAB-based bioluminescence assays **4**
Figure S2 4
Table S4 4
Table S5 5
Table S6 5
Figure S3 5

Biotransformations and chromatographic analysis **6**
Table S7 6
Figure S4 6
Figure S5 7

References **7**

Author Contributions **7**

Experimental Procedures and Additional Results

Site-directed mutagenesis of BVMO*Halo* and assembly of the biosensor plasmid pLA1

As described in detail in the main manuscript, site-directed mutagenesis targeted active site residues of BVMO*Halo* (Baeyer-Villiger monooxygenase from *Halopolyspora algeriensis*[1]) and was performed through the Q5® Mutagenesis Kit, following the instructions by the supplier (#E0554S, New England Biolabs). Primers were designed with the NEBaseChanger® v2.4.2 webtool (https://nebasechanger.neb.com/) and are summarized in Table S1.

Table S1. Primers for alanine scanning of BVMO*Halo* active site residues

Primer	Sequence (5' to 3')
V59A_fwd	ACGCTGTGAT**GCG**GAAAGTCTGA
V59A_rev	GCACCCGGATAACGATTC
R330A_fwd	ACCAAACGTC**GCG**GCCTGGATACCGGTTATTATG
R330A_rev	GCCGAACGGGAAGCTACG
T428A_fwd	TGTTCACCAT**GCC**GGTCCGAGCA
T428A_rev	GATTCGGGAAGCCTGCAATG
C144A_fwd	GGCAACCGGC**GCC**CTGAGCATGA
C144A_rev	ATAATAACGAAGCGGGCG
T46A_fwd	TGTTGGTGGT**GCC**TGGTATTGGAATC
T46A_rev	TCGGCACCGGCTTCGAAC
N50A_fwd	CTGGTATTGG**GCT**CGTTATCCGGGTG
N50A_rev	GTACCACCAACATCGGCA
Y52A_fwd	TTGGAATCGT**GCT**CCGGGTGCAC
Y52A_rev	TACCAGGTACCACCAACA
R56A_fwd	TCCGGGTGCA**GCC**TGTGATGTGG
R56A_rev	TAACGATTCCAATACCAG
C57A_fwd	GGGTGCACGC**GCT**GATGTGGAAAG
C57A_rev	GGATAACGATTCCAATACC
D58A_fwd	TGCACGCTGT**GCT**GTGGAAAGTC
D58A_rev	CCCGGATAACGATTCCAATAC
L145A_fwd	AACCGGCTGC**GCG**AGCATGAGCA
L145A_rev	GCCATAATAACGAAGCGG
S188A_fwd	TGGCACCGGC**GCT**AGTGGTATTC
S188A_rev	ATAACACCCAGACGTTTAC
Q192A_fwd	TAGTGGTATT**GCG**AGCATTCCGATTCTGG
Q192A_rev	CTGCCGGTGCCAATAACA
F382A_fwd	CGCCACCGGC**GCC**GATGCAATGA
F382A_rev	AACACCAGGCTATCGAATTC
M439A_fwd	TCTGAGCAAT**GCG**ATGGTGAGTATTGAACAGC
M439A_rev	ACACTCGGACTGCTCGGA
N499A_fwd	TATGGGTAGC**GCT**GTGCCGGGTAAAC
N499A_rev	TACCAACTATCTGCGGTC
D383A_fwd	CACCGGCTTC**GCT**GCAATGACCG
D383A_rev	GCGAACACCAGGCTATCG
S498A_fwd	GTATATGGGT**GCC**AATGTGCCGGG
S498A_rev	CAACTATCTGCGGTCGGA
G381A_fwd	GTTCGCCACC**GCC**TTCGATGCAA
G381A_rev	ACCAGGCTATCGAATTCATAATCAC
W494A_fwd	CGCAGATAGT**GCG**TATATGGGTAGCAATG
W494A_rev	GTCGGATACAGGGTTGCA
S493A_fwd	GACCGCAGAT**GCT**TGGTATATGGGTAG
S493A_rev	GGATACAGGGTTGCATTAC

For the construction of pLA1 (pCDF_*luxAB::alkJ*), which combines the open reading frames (ORFs) of *luxAB* and *alkJ* on a pCDF-derived vector as described in the main article, sequence- and ligation-independent cloning (SLIC) protocols were used as reported previously.[2] The DNA fragment containing *alkJ* was amplified by polymerase chain reaction (PCR) with Pfu$^+$ and the primer pair AlkJ-CDF_F/R (Table S2). The latter also introduced a 20 bp-overhang complementary to the target pCDF backbone for subsequent assembly by SLIC. The pCDF backbone including the *luxAB*-coding region was amplified with *Opti*Taq polymerase and the primer pair pCDF_F/R (Table S2). PCR mixtures were prepared as reported previously.[2] The optimized thermal cycle conditions are given in Table S3. Purification of PCR products was performed using the NuceloSpin® Gel and PCR Clean-up Kit (MACHEREY-NAGEL GmbH & Co. KG, Dürren, Germany) as instructed by the supplier. Following our established SLIC protocols, Sanger sequencing confirmed the correct assembly of pLA1.

Table S2. Primers used for the assembly and sequencing of pLA1

Primer	Sequence (5' to 3')
AlkJ-CDF_F	CTGCAGGTCGACAAGCTTGCCGTAGAGGATCGAGATCTCG
AlkJ-CDF_R	GCAGCGGTTTCTTTACCAGATTACATGCAGACAGCTATCATGG
pCDF_F	TCTGGTAAAGAAACCGCT
pCDF_R	GCAAGCTTGTCGACCTG
SEQ-AlkJ_F	TATGTACGACTATATAATCGTTG
SEQ-AlkJ_R	TTACATGCAGACAGCTATCATGG
mSEQ-AlkJ_F	GTGCATTCTTGCACGGTG
mSEQ-AlkJ_R	GCCACGTTACTAGTGAG

Table S3. Optimized thermal cycle conditions for the assembly of pLA1

PCR step (Pfu$^+$)	Temperature [°C]	Time	No. of cycles
Initial denaturation	95	5 min	1
Denaturation	95	30 s	
Annealing	48.6	30 s	30
Extension	72	1 min 45 s	
Terminal extension	72	3 min	1
Hold	10–12	∞	1

PCR step (*Opti*Taq)	Temperature [°C]	Time	No. of cycles
Initial denaturation	95	5 min	1
Denaturation	95	30 s	
Annealing	48.6	20 s	30
Extension	72	5 min 45 s	
Terminal extension	72	7 min	1
Hold	10–12	∞	1

Protein expression and SDS-PAGE analysis

The enzymes CHMO$_{Acineto}$, BS2, AlkJ, and LuxAB were expressed in *Escherichia coli* (*E. coli*) K-12 MG1655, exhibiting reduced aromatic reduction activity, herein referred to as *E. coli* RARE.[2–4] BVMO$_{Halo}$ and corresponding variants were expressed in *E. coli* TOP10. Expression protocols are described in detail in the main article. To confirm protein production, whole-cells were analyzed by 12.5% (*ω/v*) sodium dodecyl sulfate-polyacrylamide gel electrophoresis (SDS-PAGE). SDS-PAGE was performed under reducing and denaturing conditions at 25 mA per gel, employing the Mini-PROTEAN electrophoresis system (Bio-Rad, Feldkirchen, Germany). Therefore, whole-cell samples were pre-treated as described previously.[2] Proteins were visualized after separation, incubating gels overnight in staining solution. The latter contained 1% (*ω/v*) α-cyclodextrin (CAS-No. 10016-20-3), 10% (*v/v*) ROTI®-Quant 5X concentrate (Carl Roth GmbH & Co. KG, Karlsruhe, Germany), and 5% (v/v) phosphoric acid (85%). To reduce background staining, gels were washed in ddH$_2$O for 2–4 h, occasionally exchanging the ddH$_2$O (Figure S1).

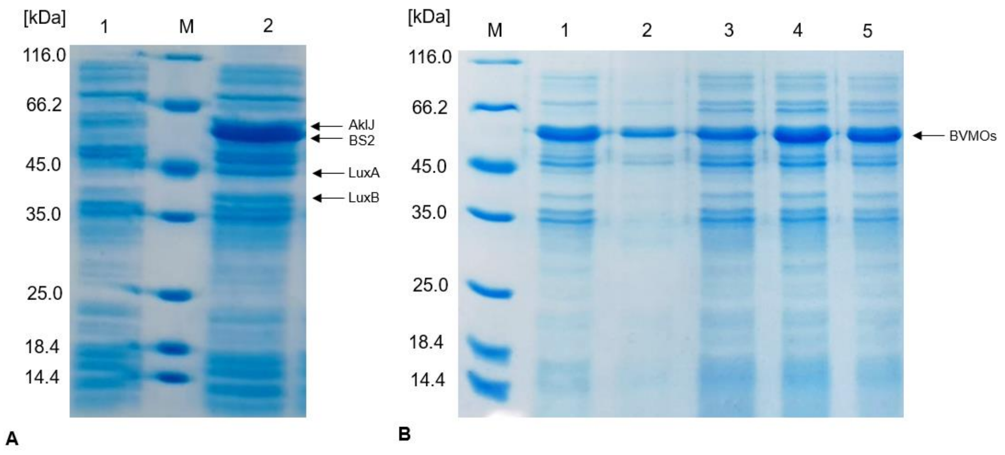

Figure S1. SDS-PAGE analysis of whole-cell samples. (**A**) Comparison of untransformed *E. coli* RARE cells (**1**) and co-expression of LuxAB [LuxA: 41 kDa, LuxB: 37 kDa] and AlkJ [61 kDa] from the newly assembled pLA1 and the esterase BS2 [54 kDa] from pET28a (**2**). The two-plasmid system (pCDFduo_*luxAB* + pACYQ_*alkJ*) was analyzed previously.[2] (**B**) Expression of BVMOs in *E. coli* TOP10: CHMO$_{Acineto}$ (**1**), BVMO$_{Halo}$ wild-type (WT; **2**), BVMO$_{Halo}$ variants C57A (**3**), S188A (**4**), and the C57A/S188A double-mutant (**5**). Sample loading normalized to OD$_{600}$ of (**A**) 5.0 and (**B**) 7.0; arrows indicate protein bands of interest. Protein marker (**M**) was loaded for size comparison.

LuxAB-based bioluminescence assays

The luciferase-based detection of aldehydes was adapted from established protocols.[2,3] Initially, resting cells (RCs) of *E. coli* RARE were used, expressing AlkJ and LuxAB from either individual plasmids (pACYQ_*alkJ* and pCDFduo_*luxAB*, respectively)[2] or the single plasmid pLA1 (see above). Functionality of the pLA1-based system was confirmed by bioluminescence produced in RCs after the addition of 1-decanol as the luciferin precursor (Figure S2). While the two-plasmid system yielded >1,600-fold increased bioluminescence within 60 min, cells harboring pLA1 produced almost 1,000-fold (984 ± 101; n = 4) increased bioluminescence and >600-fold (637 ± 132; n = 4) after 15 min and 60 min reaction time, respectively. We attribute the smaller standard deviation (SD) between biological assay replicates to the reduced metabolic burden from the maintenance and expression of two genes from only a single plasmid.[5] For bioluminescence assays employing RCs co-expressing BS2, AlkJ, and LuxB, see Figure 2 in the main article.

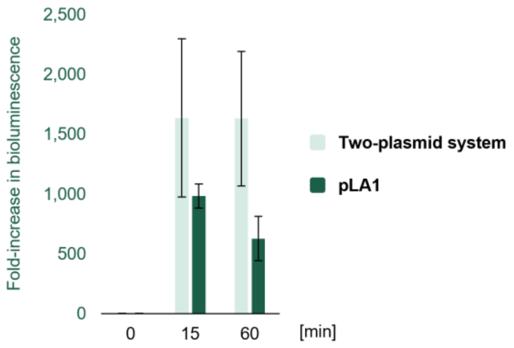

Figure S2. Functionality of pLA1. The emission of bioluminescence in response to the oxidation of 1-decanol to decanal was monitored in *E. coli* RARE RCs (OD_{600} ≈ 10.0) co-expressing AlkJ and LuxAB from two plasmids (mint; pACYQ_*alkJ* and pCDFduo_*luxAB*, respectively)[2] or the newly assembled pLA1 (dark green). Bioluminescence was recorded before (0 min) and after the addition (15–60 min) of 1 mM 1-decanol. The experimental cut-off (XCO) values were determined in the presence of 1% (*v/v*) dimethylformamide (DMF) as described in the main article. The pLA1-based system was characterized by a low bioluminescence background and yielded lower but consistent increases in bioluminescence signals over the monitoring time. Bars represent the mean fold-increase in bioluminescence above background ± SD (n = 4).

As discussed previously and in the main article, the fold-increase in bioluminescence over background is calculated by dividing the bioluminescence signal at each time point (t_x) by the background bioluminescence at t_0. In accordance to previous studies employing LuxAB as biosensor for aldehydes, the bioluminescence slightly increased in the presence of organic co-solvent (i.e., in the absence of any luciferin) and was monitored to calculate the XCO.[2] In the following, the XCO values (determined in the presence of 1% (*v/v*) DMF as co-solvent; b_{DMF}) and the increase in bioluminescence in response to the formation of aldehydes from ester compounds (**1**–**11b**) and primary alcohols (**1**–**11c**) are summarized for the newly assembled pLA1 (Table S4 and Table S5, respectively; n ≥ 3). Importantly, supplementation of the ketones (**1**–**11a**) to RCs – co-expressing BS2, AlkJ and LuxAB as before – did not increase the bioluminescence above background (Table S6). This proves that unreacted ketones, for example, from previous BVMO reactions do not interfere with the biosensor system. LuxAB-based assays were performed as before (see Figure S2).

Table S4. Determination of the XCO value and mean fold-increase in bioluminescence ± SD for ester compounds (**1**–**11b**) used in this work; n.a. = not available.

Time (min)	b_{DMF}	XCO	1b	2b	3b	4b	5b
0	1.0 ± 0.4	1.4	1.0 ± 0.4	1.0 ± 0.5	1.0 ± 0.5	1.0 ± 0.5	1.0 ± 0.5
3	5.4 ± 2.5	7.9	3.6 ± 1.7	28.7 ± 12.3	316.2 ± 152.1	85.7 ± 49.7	361.5 ± 81.0
6	5.0 ± 2.5	7.5	7.5 ± 2.4	40.6 ± 15.1	226.5 ± 100.8	132.4 ± 57.4	183.6 ± 8.2
9	4.5 ± 2.4	6.8	7.4 ± 2.1	39.2 ± 14.1	227.6 ± 95.4	140.7 ± 60.5	130.4 ± 0.4
12	2.7 ± 1.4	4.1	5.1 ± 1.3	25.7 ± 9.2	170.0 ± 85.1	113.8 ±50.1	130.4 ± 0.4
15	2.4 ± 1.1	3.5	4.3 ± 1.1	22.2 ± 7.4	165.8 ± 77.1	134.6 ± 58.3	99.2 ± 3.7

Time (min)	6b	7b	8b	9b	10b	11b
0	1.0 ± 0.3	1.0 ± 0.4	1.0 ± 0.2	1.0 ± 0.1	n.a	1.0 ± 0.1
3	266.3 ± 82.0	47.2 ± 11.8	47.2 ± 11.8	29.7 ± 3.4	n.a	1.7 ± 0.1
6	224.4 ± 65.5	106.6 ± 23.4	106.6 ± 23.4	53.8 ± 7.9	n.a	1.6 ± 0.1
9	212.1 ± 54.0	161.6 ± 26.0	161.6 ± 26.0	82.2 ± 7.8	n.a	1.6 ± 0.1
12	167.0 ± 54.5	124.3 ± 28.8	124.3 ± 28.8	78.4 ± 12.3	n.a	1.0 ± 0.0
15	168.8 ± 49.5	156.6 ± 28.2	1056.6 ± 28.2	106.2 ± 10.9	n.a	1.0 ± 0.0

Table S5. Determination of the XCO value and mean fold-increase in bioluminescence ± SD for alcohol compounds (**1–11c**) used in this work.

Time (min)	b_{DMF}	XCO	1c	2c	3c	4c	5c	6c
0	1.0 ± 0.1	1.1	n.a	1.0 ± 0.1	1.0 ± 0.1	n.a	1.0 ± 0.1	1.0 ± 0.1
3	19.2 ± 7.4	26.6	n.a	93.6 ± 23.3	87.5 ± 37.5	n.a	977.2 ± 87.2	848.4 ± 173.9
6	9.0 ± 9.9	18.8	n.a	162.2 ± 20.1	108.4 ± 67.8	n.a	507.8 ± 36.7	547.4 ± 46.9
9	6.4 ± 7.1	13.5	n.a	153.6 ± 29.3	115.0 ± 88.8	n.a	469.0 ± 28.9	460.6 ± 98.7
12	6.0 ± 6.3	12.2	n.a	149.5 ± 41.9	122.8 ± 95.6	n.a	474.5 ± 34.4	426.4 ± 90.3
15	4.6 ± 4.9	9.5	n.a	140.4 ± 65.0	197.3 ± 15.8	n.a	415.5 ± 36.4	368.6 ± 82.2

Time (min)	7c	8c	9c	10c	11c
0	1.0 ± 0.1	1.0 ± 0.1	1.0 ± 0.0	1.0 ± 0.0	1.0 ± 0.1
3	588.0 ± 108.4	413.9 ± 157.8	199.8 ± 21.1	190.7 ± 21.0	44.0 ± 7.7
6	517.8 ± 57.0	414.2 ± 147.9	252.4 ± 3.8	197.8 ± 8.9	15.4 ± 1.8
9	390.4 ± 51.2	309.8 ± 97.1	212.9 ± 7.9	159.2 ± 9.4	8.9 ± 0.9
12	376.9 ± 46.4	306.2 ± 90.9	223.5 ± 8.7	158.5 ± 12.2	6.9 ± 0.7
15	312.4 ± 35.3	256.8 ± 72.9	180.5 ± 8.7	128.3 ± 10.9	5.0 ± 0.5

Table S6. Determination of the XCO value and mean fold-increase in bioluminescence ± SD for ketone compounds (**1–11a**) used in this work.

Time (min)	b_{DMF}	XCO	1a	2a	3a	4a	5a
0	1.0 ± 0.7	1.7	1.0 ± 0.1	1.0 ± 0.1	n.a.	1.0 ± 0.1	1.0 ± 0.1
3	5.5 ± 0.6	6.1	2.7 ± 0.4	5.0 ± 0.6	n.a.	11.8 ± 2.3	3.7 ± 0.7
6	26.8 ± 8.7	35.5	10.2 ± 3.4	12.0 ± 2.9	n.a.	17.7 ± 5.9	13.6 ± 3.1
9	33.8 ± 7.8	41.6	13.6 ± 4.8	14.0 ± 3.1	n.a.	17.2 ± 6.1	20.8 ± 6.0
12	33.4 ± 3.3	36.6	18.1 ± 6.0	21.1 ± 4.5	n.a.	18.3 ± 6.1	28.3 ± 12.1
15	34.2 ± 1.5	35.6	21.3 ± 6.0	25.9 ± 6.4	n.a.	19.1 ± 8.1	31.9 ± 14.2

Time (min)	6a	7a	8a	9a	10a	11a
0	1.0 ± 0.2	n.a.	1.0 ± 0.1	1.0 ± 0.0	n.a.	1.0 ± 0.1
3	2.0 ± 0.4	n.a.	3.6 ± 0.6	3.3 ± 0.1	n.a.	2.7 ± 0.4
6	1.7 ± 0.4	n.a.	21.1 ± 2.0	20.0 ± 6.0	n.a.	10.4 ± 0.5
9	1.1 ± 0.2	n.a.	30.4 ± 1.2	34.8 ± 15.9	n.a.	13.1 ± 1.4
12	0.8 ± 0.2	n.a.	38.5 ± 7.1	45.3 ± 22.8	n.a.	14.6 ± 2.8
15	0.9 ± 0.2	n.a.	37.9 ± 11.6	50.6 ± 27.8	n.a.	16.4 ±5.4

For the screening of BVMO*Halo* variants, either cell-free extracts (CFEs; Figure S3) or RCs of *E. coli* TOP10 containing the desired mutant were analyzed by LuxAB-based assays as described in the main article (Figure 4A).

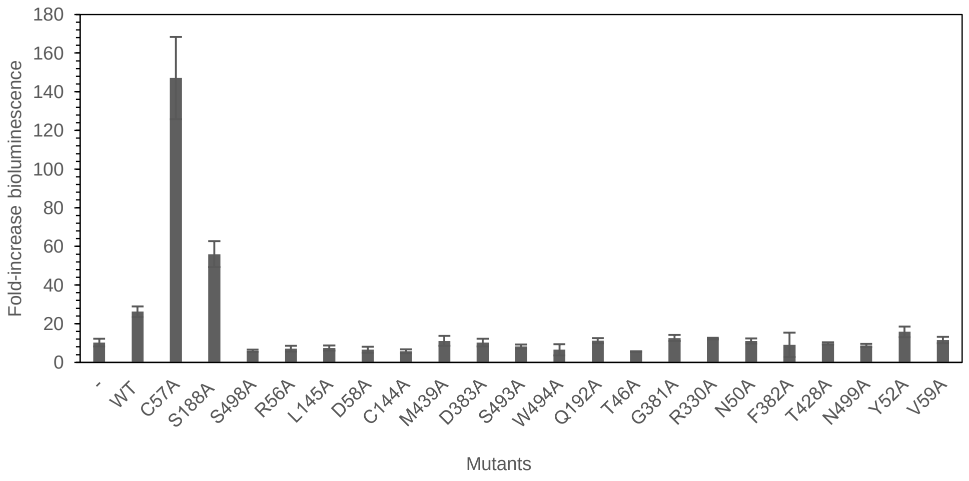

Figure S3. LuxAB-based screening of enzyme variants *in vitro*. Supernatants potentially containing the reaction product *n*-decyl acetate (**6b**) from reactions with the indicated BVMO*Halo* mutant and 2-dodecanone substrate (**6a**) were prepared as described in the main article. Reaction mixtures were added to RCs of *E. coli* RARE (OD_{600} ≈ 10.0), co-expressing BS2 (pET28a), AlkJ and LuxAB (pLA1). The bioluminescence was monitored. Bioluminescence signals represented as mean fold-increases + SD after 15 min incubation time of independently performed BVMO reactions (n ≥ 2). CFEs were not normalized in respect to the total amount of protein, for example. CFEs produced from *E. coli* TOP10 cells harboring the empty pBAD vector were used as negative control (–); wild-type BVMO*Halo* (WT) was used as the positive control.

Biotransformations and chromatographic analysis

For whole-cell biotransformations, *E. coli* RCs containing the desired BVMO were prepared and adjusted to an OD_{600} ≈ 10.0 in resting cell medium (RCM; 22 mM KH_2PO_4, 42 mM Na_2HPO_4, 8.56 mM NaCl, 1 mM $MgSO_4$, 0.1 mM $CaCl_2$, and 1% (*ω/v*) glucose) as described in the main article. Ketone substrates were added to a final concentration of 5 mM. Reactions were performed in glass vials, incubated at 25°C with shaking (220 rpm; INFORS HT Multitron) for 24 h. For GC/FID analysis, samples (100 µL) were extracted two times with 200 µL ethyl acetate containing 1 mM methyl benzoate as internal standard (IS) as described in the main article. Reaction compositions were analyzed on a GC-2010 Plus (Shimadzu), equipped with a ZB5MSi column (length: 30 m; inner diameter: 0.25 mm; film thickness: 0.25 µm) from Phenomenex (Torrance, USA), a flame ionization detector (FID), and an auto-injector (Shimadzu). Separation used GC/FID methods reported previously.[2] For quantification, the relative response factors (RFFs) of investigated compounds were determined as described in the main article and are summarized in Table S7. Time-resolved conversion of the benchmark ketone **6a** (2-dodecanone) are shown in Figure S4. Cyclohexanone monooxygenase from *Acinetobacter* sp. ($CHMO_{Acineto}$) was used as additional control for the oxidation of **6a** and cyclohexanone; oxidation of the latter yielded ε-caprolactone (Figure S5).[6]

Table S7. Compounds analyzed by GC/FID

#	Compound	RFF
**5a**	2-undecanone	1.151
**5b**	*n*-nonyl acetate	0.534
**6a**	2-dodecanone	1.285
**6b**	*n*-decyl acetate	0.655
-	cyclohexanone	1.234
-	ε-caprolactone	0.446
IS	methyl benzoate	-

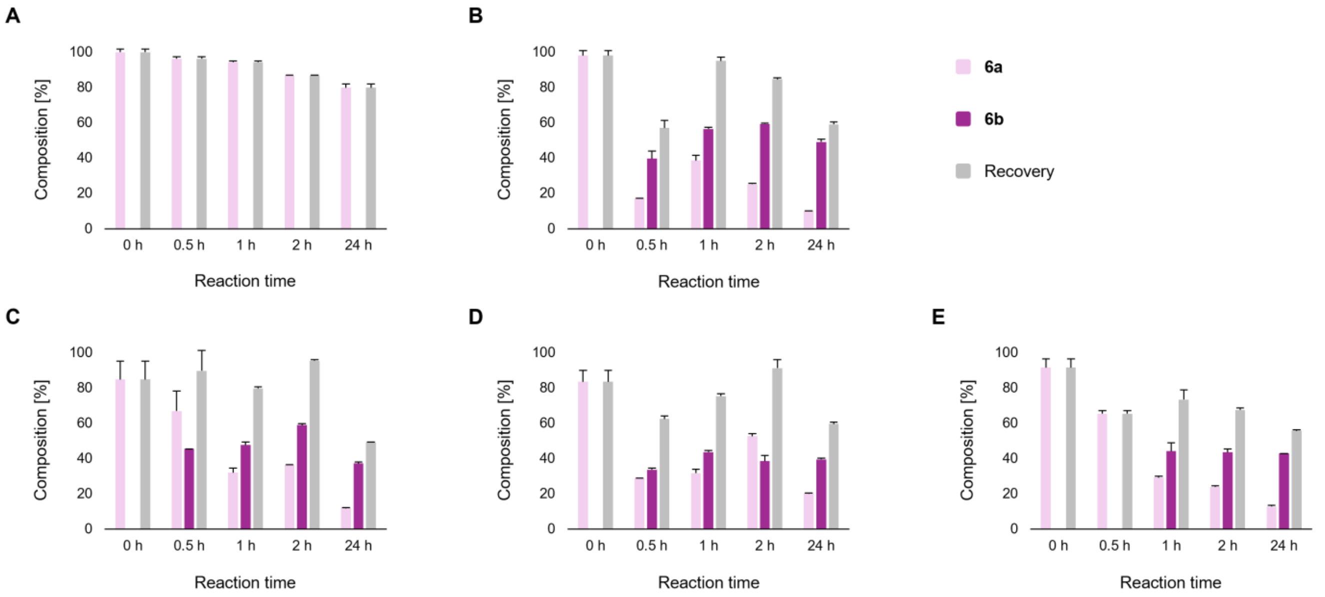

Figure S4. Production of the ester 6b by $BVMO_{Halo}$ and selected variants. Oxidation of **6a** (light pink) into **6b** (magenta) in RCs of *E. coli* TOP10 (OD_{600} ≈ 10.0) expressing (**A**) no BVMO (*E. coli* TOP10 transformed with empty pBAD), (**B**) $BVMO_{Halo}$ WT, (**C**) $BVMO_{Halo}$ C57A, (**D**) $BVMO_{Halo}$ S188A, and (**E**) $BVMO_{Halo}$ C57A/S188A. Biotransformations were performed at 5 mM substrate load in the presence of 5% (*v/v*) DMF as co-solvent (25°C, 200 rpm, 24 h). The composition of reaction mixtures and the recovery of material [%] are represented as mean values + SD of biological replicates (n ≥ 2) based on calibrated GC/FID analysis. Reduced recoveries attributed to low solubility and/or volatility of compounds, for example.

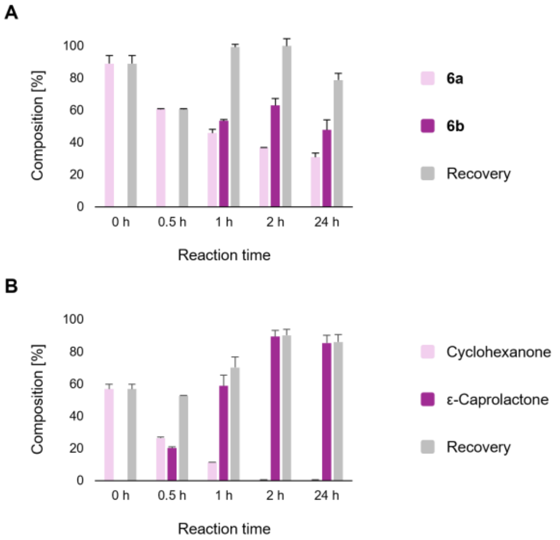

Figure S5. Activity of CHMO*Acineto* *in vivo*. Oxidation of (**A**) **6a** into **6b** and (**B**) cyclohexanone into ε-caprolactone by CHMO*Acineto*. Biotransformations employed RCs of *E. coli* RARE (OD_{600} ≈ 10.0) expressing the BVMO from pET28a and 5 mM ketone substrate. Reactions were carried out in the presence of 5% (*v/v*) DMF as co-solvent at 25°C with shaking (180 rpm) for 24 h. The composition of reaction mixtures and the recovery of material [%] are represented as mean values + SD of biological replicates (n ≥ 2) based on calibrated GC/FID analysis. Reduced recoveries attributed to low solubility and/or volatility of compounds, for example.

References

[1] T. Sakoleva, H. P. Austin, C. Tzima, M. Dörr, U.T. Bornscheuer, *ChemBioChem* **2023**, *24*, e202200746.
[2] T. Bayer, A. Becker, H. Terholsen, I. J. Kim, I. Menyes, S. Buchwald, K. Balke, S. Santala, S.C. Almo, U.T. Bornscheuer, *Catalysts* **2021**, *11*, 953.
[3] T. Bayer, L. Pfaff, Y. Branson, A. Becker, S. Wu, U.T. Bornscheuer, R. Wei, *iScience* **2022**, *25*, 104326.
[4] A.M. Kunjapur, Y. Tarasova, K.L.J. Prather, *J. Am. Chem. Soc.* **2014**, *136*, 11644–11654.
[5] T. Bayer, S. Milker, T. Wiesinger, F. Rudroff, M.D. Mihovilovic, *Adv. Synth. Catal.* **2015**, *357*, 1587–1618.
[6] S. Schmidt, U. T. Bornscheuer, *The Enzymes* (Eds: P. Chaiyen, F. Tamanoi), Elsevier, **2020**, pp. 231–281.

Author Contributions

Conceptualization: T.B.; Data curation: T.B., M.D., L.K., T.S., J.E.S., F.V.; Formal analysis: T.B., L.K., T.S., J.E.S., F.V.; Funding acquisition: T.B., U.T.B.; Investigation: T.B., S.G., L.K., K.D.N., T.S., J.E.S., F.V.; Methodology: T.B.; Project administration: T.B., U.T.B.; Resources: T.B., U.T.B.; Supervision: T.B., U.T.B., M.D., S.G.; Validation: T.B., K.D.N., T.S., S.G.; Visualization: T.B., L.K., T.S., J.E.S.; Writing – original draft: T.B., T.S.; Writing – review & editing: all authors.
